# Variation in the gene coding for the M5 Muscarinic receptor (*CHRM5*) influences cigarette dose but is not associated with dependence to drugs of addiction: evidence from a prospective population based cohort study of young adults

**DOI:** 10.1186/1471-2156-8-46

**Published:** 2007-07-03

**Authors:** Richard JL Anney, Mehrnoush Lotfi-Miri, Craig A Olsson, Sophie C Reid, Sheryl A Hemphill, George C Patton

**Affiliations:** 1 Neuropsychiatric Genetics Research Group, Department of Psychiatry, Trinity College Dublin, Dublin, Ireland; 2 Behavioural Genetics Laboratory, Murdoch Childrens Research Institute, Parkville, Victoria, Australia; 3 Centre for Adolescent Health, Murdoch Childrens Research Institute, Parkville, Victoria, Australia; 4 Department of Paediatrics, University of Melbourne, Melbourne, Victoria, Australia

## Abstract

**Background:**

The mesolimbic structures of the brain are important in the anticipation and perception of reward. Moreover, many drugs of addiction elicit their response in these structures. The M5 muscarinic receptor (M5R) is expressed in dopamine-containing neurones of the substantia nigra pars compacta and ventral tegmental area, and regulates the release of mesolimbic dopamine. Mice lacking M5R show a substantial reduction in both reward and withdrawal responses to morphine and cocaine. The *CHRM5*, the gene that codes for the M5R, is a strong biological candidate for a role in human addiction. We screened the coding and core promoter sequences of *CHRM5* using denaturing high performance liquid chromatography to identify common polymorphisms. Additional polymorphisms within the coding and core promoter regions that were identified through dbSNP were validated in the test population. We investigated whether these polymorphisms influence substance dependence and dose in a cohort of 1947 young Australians.

**Results:**

Analysis was performed on 815 participants of European ancestry who were interviewed at wave 8 of the cohort study and provided DNA. We observed a 26.8% increase in cigarette consumption in carriers of the rs7162140 T-allele, equating to 20.1 cigarettes per week (p=0.01). Carriers of the rs7162140 T-allele were also found to have nearly a 3-fold increased risk of developing cannabis dependence (OR=2.9 (95%CI 1.1-7.4); p=0.03).

**Conclusion:**

Our data suggest that variation within the *CHRM5 *locus may play an important role in tobacco and cannabis but not alcohol addiction in European ancestry populations. This is the first study to show an association between *CHRM5 *and substance use in humans. These data support the further investigation of this gene as a risk factor in substance use and dependence.

## Background

The muscarinic acetylcholine M5 receptor (M5R) is a member of the family of G-protein coupled receptors. The M5R is the only muscarinic receptor subtype expressed in the ventral tegmetal area (VTA) and substantia nigra pars compacta (SNc) [1-3], where it is co-localised with dopamine D2 receptors on dopaminergic neurones [1]. There is considerable evidence to suggest that stimulation of dopaminergic pathways within the VTA and SNc gives rise to the subjective experience of pleasure and reward.

Drugs of addiction, such as tobacco, alcohol, cannabis, cocaine and opiates, are thought to exert their reinforcing effects by elevating dopamine levels within the mesolimbic structures [4]. While the exact biological mechanisms that determine substance-driven reward are poorly understood, genes involved in the regulation of dopaminergic neurotransmission are thought to be key components in this process. The M5R is believed to modulate dopaminergic neurotransmission within VTA and SNc dopamine neurones [5]. Specifically, the M5R is thought to control the duration of dopamine release [1,6,7].

Animal models show that the M5R plays an important role in modulating substance addiction. Mice lacking a functional M5R were shown to have reduced reward and withdrawal responses following morphine [8] and cocaine [9] administration. Tobacco, alcohol and cannabis exert their reinforcing effects through similar dopaminergic mechanisms to cocaine and opiates [4]. Consequently, the gene coding for the human M5R, *CHRM5 *(GeneID: 1133) is a strong biological candidate for studies looking at genetic influence on addiction to tobacco, alcohol and cannabis.

The purpose of this study was two-fold. Firstly, to screen the complete coding, untranslated and putative "core" promoter regions of *CHRM5 *for common polymorphic variation. Secondly, to assess the association between identified polymorphic loci and drug dependence in a large cohort of young adults. To our knowledge this is the first study to report a systematic screening for polymorphisms within the *CHRM5 *locus. Consequently, there have been no association studies investigating the contribution of natural genetic variation at this locus to risk of substance dependence.

## Results

### Analysis of the *CHRM5 *locus

**Figure 1:**
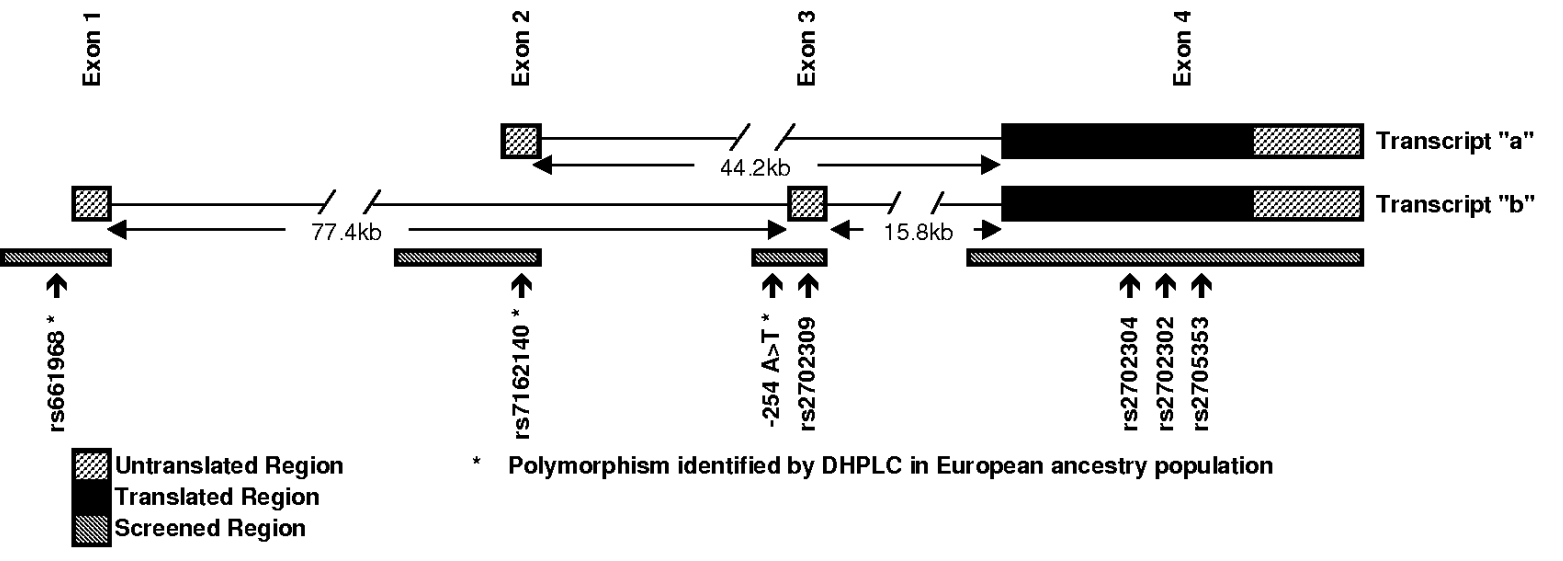
Illustration of the *CHRM5 *gene locus on chromosome 15q26. The two transcripts were defined from the cDNA sequences AB084282 (transcript *CHRM5*. a) and AK095198 (transcript *CHRM5*. b). Exons are shown as blocks along the genomic sequence. The intronic region has been abridged for the purposes of this illustration.

*CHRM5 *(LocusID: 1133), the gene that codes for the M5R, is located at 15q26. *In silico *analysis of the *CHRM5 *locus identified two mRNA species, *CHRM5*. a (described by accession number: AB084282,) and *CHRM5*. b (described by accession number: AK095198). The gene is alternatively spliced with two transcripts *CHRM5*. a and *CHRM5*. b containing a common 3'-coding exon and three alternatively spliced 5'-untranslated exons (see Figure 1). Both species contain the coding exon 4 with alternative splicing in the 5'untranslated region. Alignment of *CHRM5*. a onto chromosome 15 reference sequence reveals a gene of two exons; a 5'-untranslated exon of 244 bp (exon 2), separated by an intron of 44.2 kbp from a 2.4 kbp exon (exon 4) containing the entire coding sequence. The *CHRM5*. b comprises of two 5'-untranslated exons of 263 bp (exon 1) and 332 bp (exon 3) respectively separated from each other and exon 4 by intronic regions of 77.4 kbp and 15.8 kbp respectively. An illustration of this gene structure is shown in Figure 1.

### Identification of polymorphic variation in the *CHRM5 *locus

Using denaturing high-performance liquid chromatography (DHPLC), we screened 4588 bp of genomic sequence of *CHRM5*, including all of the sequence represented in the final mRNA and both core promoter regions for the transcripts *CHRM5*. a and *CHRM5*. b. The chosen screening regions are highlighted on Figure 1. We identified three polymorphisms by this method, rs661968, rs7162140 and *CHRM5*b*-257A>T. We determined allele frequencies of the polymorphic SNPs in a subset of our test population; minor allele frequencies were 3.2% (rs661986), 19.0% (rs7162140) and 2.8% (*CHRM5*b. -257A>T). In the course of this study rs661968 and rs7162140 were described in the public sequence variation (dbSNP) database. Analysis of our screening region within the dbSNP database identified four polymorphisms not detected by DHPLC in our screening region (rs2702309, rs2702304, rs2576302 and rs2705353). None of the four variants identified were polymorphic in our test population.

### Association of rs7162140 and substance dependence

Of the seven polymorphisms identified within our screening region only rs7162140 was of sufficient frequency for association analysis. We examined nicotine dependence, tobacco dose, alcohol dependence, alcohol dose and cannabis dependence. A summary of genotype association data for dependence measures are shown in Table 1.

**Table 1:** Genotype associations between rs7162140 and categorical measures of substance dependence.

	Genotype Count				
	Case	Control	Model	Odds Ratio	p-Value
Nicotine Dependence					
CC	48	144	Dominant	1.6 (0.90–2.8)	0.11
CT	24	49	Recessive	2.6 (0.65–11.0)	0.17
TT	4	4			

Alcohol Dependence					
CC	310	50	Dominant	1.3 (0.77–2.0)	0.36
CT	135	28	Recessive	0.95 (0.27–3.3)	0.94
TT	18	3			

Cannabis Dependence					
CC	40	19	Dominant	2.9 (1.1–7.4)	0.03
CT	9	13	Recessive	1.5 (0.20–11.4)	0.68
TT	2	2			

Odds ratio shown with 95% confidence intervals. Analysis based on both dominant (TT|CT > CC) and recessive (TT > CT|CC) inheritance models.

#### Tobacco use

For tobacco use behaviours, we found no evidence of susceptibility to nicotine dependence with respect to the rs7162140 T-allele under either the dominant or recessive model. Interstingly, we observed a substantial increase in tobacco dose (dominant model) among carriers of the T-allele. Carriers of the T-allele smoked on average 20.1 cigarettes more per week than individuals who were homozygous for the C-allele (CC = 74.9 cpw (SEM 4.0), CT|TT = 95.0cpw (SEM 7.6)), (nominal p = 0.01) (see Table 2).

**Table 2:** Genotype association between rs7162140 and quantitative measures of substance dependence.

	n	Mean	SD	Model	p-Value (two-tailed)
Tobacco Dose					
CC	192	74.9	55.1	Dominant	0.01
CT	73	97.0	70.9	Recessive	0.84
TT	8	76.6	35.8		
CT|TT	81	95.0	68.4		

Alcohol Dose					
CC	354	20.1	21.8	Dominant	0.81
CT	161	21.1	21.1	Recessive	0.41
TT	21	16.4	16.2		
CT|TT	182	20.6	20.7		

Alcohol dose described as units (10 g) per week. Tobacco dose described as cigarettes smoked per week. Analysis based on both dominant (TT|CT > CC) and recessive (TT > CT|CC) inheritance models using the Student's t-test (two-tailed) to measure equality of means.

#### Alcohol use

We did not find any evidence to support a role of rs7162140 and alcohol dose or dependence (see Table 1 and Table 2).

#### Cannabis use

Genotype analysis revealed a nearly three-fold increased risk observed under a T-allele dominant model (Odds ratio = 2.9 (95%CI 1.1–7.4), nominal p = 0.03) (see Table 1).

## Discussion

This study reports a systematic screen of the known transcribed and core promoter regions of the human *CHRM5*. We identified seven variants within the screening regions, three by DHPLC and four additional variants through public sequence variation repositories. Only polymorphisms identified by DHPLC were polymorphic in our population. Of all of the markers that were polymorphic within the Australian Caucasian population, only rs7162140 was deemed to have a mior allele frequency of sufficient magnitude to be examined in our population.

Our results support a role for the rs7162140 T-allele in risk of cannabis dependence and tobacco dose. We observed a 26.8% increase in cigarette consumption in carriers of the T-allele, equating to 20.1 cigarettes per week (nominal p = 0.01). Therapeutic intervention leading to a reduction in tobacco consumption at this level would have considerable public health implications. Carriers of the T-allele were also found to have an increase in their risk of cannabis dependence by nearly 3-fold. These data complement animal models that have shown the M5R is important in modulating the reward and withdrawal processes associated with opiate and cocaine use. We did not observe association between measures of alcohol dependence or dose. Although our data for tobacco and cannabis fit a dominant mode of inheritance, we did not have sufficient power to distinguish between the dominant and heterosis-mode of inheritance for this allele. Moreover, we would exercise caution in the over interpretation of these data. We have not applied correction for multiple testing and therefore would support further studies to confirm or reject these findings.

One strength of this study is the theory-driven approach to candidate gene selection. From a purely biological perspective, the M5R is a strong candidate through its role in the control of the duration of dopamine release [1,6,7]. Mice lacking a functional M5R have reduced reward and withdrawal responses following morphine [8] and cocaine [9] administration. We examined the role of variation in the *CHRM5 *gene and dependence on three drugs of addiction, namely tobacco, alcohol and cannabis. The dose and dependence variables that we have examined are derived from phenotypic constructs that are common to other studies such that cross-study investigation can be facilitated.

We have attempted to improve the probability of identifying causative genetic loci that influence risk of drugs of addiction by hypothesis-based candidate polymorphism choice. Since it is not feasible to screen and study the entire genomic sequence within the gene locus, we chose to employ the VAPSE (or Variants Affecting Protein Structure or Expression) approach [10], whereby analysis is restricted to gene sequences coding for domains which are likely to affect protein structure or expression. Under the VAPSE paradigm, we predict that any functional variation is measured directly and therefore we exclude confounding associated with linkage disequilibrium. However, as a precaution analysis was restricted to individuals of European ancestry to lessen genetic and non-genetic confounders specific to ethnicity. The rs7162140 polymorphism is transcribed in the *CHRM5 *message. The polymorphism is located within the 5' untranslated region of *CHRM5*. The specific function of this polymorphism on gene expression or function is unknown and requires further investigation. We acknowledge that our approach and chosen screening region cannot account for the influence of long-range regulatory elements or that of epigenetic modifications that alter the function of the *CHRM5 *gene. However, consistent with our study design, a recent report indicates that polymorphisms that alter gene expression are strongly biased towards the core and proximal promoter elements [11].

Studies on rats deficient in M5R have shown that midbrain M5R mediate the duration of forebrain dopamine transmission and the maintenance of dopamine-related reward [6,7]. Moreover, mice lacking a functional M5R were shown to have reduced reward and withdrawal responses following morphine [8] and cocaine [9] administration. M5R -/- mice show reduced self administration of cocaine compared to M5R +/+ controls [9]. Our data show that polymorphisms within the *CHRM5 *gene locus increase tobacco use, suggesting increased reward and withdrawal following substance use in the subset containing the rs7162140 T-allele. Combined with data from animal models we would hypothesise that the rs7162140 T-allele, or an allele in linkage disequilibrium enhances the functional capacity of the M5R compared to the C-allele. Daily tobacco dose is associated with nicotine dependence in our sample (Student's T-test; mean cigarettes per week of current smokers, subsyndromal nicotine dependent = 55.7, nicotine dependent = 129.9; p < 0.0001). We observe a statistically non-significant increase in nicotine dependence in this sample (Dominant model OR = 1.6 (95%CI 0.9–2.8). One reason for the lack of association for the categorical dependence traits may be the limitation of sample size (nicotine dependence n = 273, cannabis dependence n = 85).

Our overall hypotheis was that *CHRM5 *would influence tobacco, alcohol and cannabis dependence. Our data does not support the role of *CHRM5 *as a generalised risk factor for drug dependence. Instead our data suggest specific effects for tobacco and cannabis but not alcohol. One explanation for this phenomenon can be drawn from the proposed action of the nicotine, alcohol and cannabis within the mesolimbic system. In the mesolimbic system, both nicotine (the active-compound in tobacco) and delta9-tetrahyrocannabinol (delta9-THC) (the active-compound in cannabis) act predominantly through receptor-mediated pathways. Specifically, nicotine operates through activation of nicotinic acetylcholine receptor (nAChR) complexes; delta9-THC operates through activation of the neural cannabinoid receptor, CB1R. As with M5R, the nAChR are co-expressed on dopamine neurones of the SNc and VTA [12]. The CB1R are present in the VTA and are co-expressed with dopamine neurones of the nucleus accumbens [13]. Interestingly, CB1R knockout mice show similar reduced reward and withdrawal to M5R knockout mice [14]. Clearly, the role of nicotine and delta9-THC in dopaminergic neurotransmission is complex, with receptors also expressed on neurones that are afferent to the mesolimbic dopamine-containing neurones. However, the co-localisation and direct ligand action on dopaminergic neurones is common to nicotine and delta9-THC. Conversely, although ethanol administration stimulates neurones within the VTA, ethanol is thought to elicit its effect through a non-ligand interaction with receptor proteins on mesolimbic-afferent neurones containing GABA_A _(gamma-aminobutyric acid type-A) and NMDA (N-methyl-D-aspartate) receptors [4]. This mode of action, specifically the predominant role of ethanol in neurones afferent to the mesolimbic dopamine/M5R-containing neurones may in part explain the lack of association seen between *CHRM5 *and alcohol dependence and dose in this study.

In addition to the role in substance dependence, *CHRM5 *may also be important in other psychiatric disorders where dysregulation of dopaminergic neurotransmission has been implicated. A recent study examined the role of *CHRM5 *in schizophrenia. The marker rs623941, located within intron 3, 2.3 kb 5' of exon 4, was not associated with schizophrenia [15]. A second marker in the alpha7-cholinergic nicotinic acetylcholine receptor (*CHRNA7*) locus was not linked with schizophrenia. When examined together the authors reported linkage with schizophrenia. However, the *CHRM5-CHRNA7 *two-marker haplotype extends over 3 Mb of genomic DNA and is unlikely to describe an ancestral chromosome block. Consequently, the linkage of the *CHRM5-CHRNA7 *haplotype with schizophrenia is unlikely to represent evidence due to linkage disequilibrium with functional markers in either gene.

## Conclusion

Animal models clearly show a role of the M5R in modulating dependence to drugs of addiction. Interestingly, herbal extracts containing known muscarinic receptor antagonists – such as scopolamine – have already been documented as useful therapies for opium addiction [16]. We show that *CHRM5 *the gene that codes for the M5R contains variations that influence the risk of substance dependence and dose in a population of young adults. Our data is the first to show an association between this gene and substance behaviours in humans and should be investigated further as potential pharmacogenetic target in the treatment in addiction.

## Methods

### Study participants

#### Screening population for polymorphism detection and validation

A screening sample for polymorphism detection and validation was drawn from a population of adolescents in the state of Victoria, Australia (Health In Transition Survey, 1994–2000) [17]. The screening sample was restricted to European Caucasian ancestry to avoid confounding by ethnicity. Fifteen individuals from the screening population were used to identify common polymorphisms in *CHRM5*.

### Cohort population for association analysis

Association between substance use behaviour and *CHRM5 *was investigated using longitudinal data from a population-based cohort of an initial potential sample of 2032 adolescents in Victoria, Australia. Participants were followed from the age of 14 to 24 years (Victorian Adolescent Health Cohort (VAHCS), 1992-present). The sample was defined in a two-stage procedure. Firstly, 45 schools were selected from a stratified frame of Government, Independent, and Catholic schools with a probability proportional to the number of Year 9 (mid-secondary school) students in each school stratum in the state. A single intact class was selected at random from each of these schools to constitute the wave one sample. At the second wave of data collection, six months later, a second intact class from each participating school was selected at random. A total of 44 schools were included in the study (24 Government, 11 Catholic and 9 Independent private schools). The first six waves of data collection were undertaken at 6-monthly intervals between 1992 and 1996. The last two survey waves were in 1998 and 2002. The survey was designed to allow DSM-IV and ICD-10 diagnoses of common mental health and behavioural problems. Among a range of psychosocial variables, the VAHCS has repeated measures on tobacco use. DNA was collected from all consenting participants. The VAHCS has maintained high participant retention with an average response rate of 80% (min 75%, max 86%). 960 participants of the 1517 participants who were interviewed in wave-8 provided DNA for genetic analysis (DNA response rate = 63.3%). For each wave of data collection, ethics approval has been provided by the Royal Children's Hospital Ethics in Human Research Committee, Melbourne, Australia.

All genetic analyses were restricted to individuals of European descent defined by country of birth across three generations (participants, parents, grandparents). "European ancestry" included individuals from non-European nations populated by predominantly European Caucasian migration (i.e. Australia, New Zealand, Canada and the United States of America). There was no significant bias in wave-8 participants who consented to provide DNA for this study compared those who did not consent. Genotyping was performed and analysed on a European ancestry cohort of 815 participants.

### Phenotype definition

Substance use was described in current users within the VAHCS participants using data from the most recent survey (wave 8). Tobacco dose, nicotine dependence, alcohol dose and alcohol dependence were defined within VAHCS participants who reported use of the drug within the week prior to the survey. Five hundred and thirty one participants reported current tobacco use (n = 273 with DNA) and 976 reported current alcohol use (n = 536 with DNA).

Cannabis use was defined as reporting use in the fortnight prior to the wave 8 survey, with current cannabis use reported in 176 participants (n = 85 with DNA). Substance dependence was described using a binary case-comparison model. Substance dose was measured according to quantitative measures.

Nicotine dependence was measured using the Fagerstrom Test for Nicotine Dependence (FTND) [18] in current smokers. Nicotine dependence was classified as reporting a FTND score equal to or greater than 4. Subsyndromal nicotine dependence was defined as scoring between one and three on the FTND; 147 participants met criteria for nicotine dependence (with DNA = 76) compared to 384 for subsyndromal-nicotine dependent controls (with DNA = 197). Alcohol and cannabis dependence was defined using the Composite International Diagnostic Interview 2.1, 12-month version (CIDI) [19]; 163 participants met criteria for alcohol dependence (with DNA = 81) compared to 813 non-alcohol dependent controls (with DNA = 463); 75 participants met criteria for cannabis dependence (with DNA = 34) compared to 101 non-cannabis dependent controls (with DNA = 51). CIDI enables a DSM-IV diagnosis of dependence, which required evidence that within the previous twelve months three of the following seven criteria must be met; (1) tolerance to the substance; (2) symptom of withdrawal upon cessation or reduced use; (3) substance used in larger amounts or for periods longer than intended; (4) persistent desire or inability to reduce or cease use; (5) a disproportionate amount of time in activities related to obtaining, using and recovering from use; (6) a significant reduction in social, recreational and occupational activities owing to substance use; and (7) continued use despite knowledge of the physical and psychological problems induced by the substance [20].

Quantitative measures of tobacco and alcohol dose were obtained using a substance use diary for the week prior to the wave 8 interviews (tobacco dose: cigarettes per week (n = 531 (273 with DNA)); alcohol dose: units (10 g) of alcohol per week (n = 962 (536 with DNA)). The variability in cannabis preparation, quality and administration precluded the administration of a quantitative variable of cannabis dose in our cohort.

#### *In Silico *analysis of the *CHRM5 *locus

The gene structure of *CHRM5 *was described through AceView, based on Build 35.1 data [21]. Specifically, gene structure was reconstructed by co-alignment of publicly available mRNA and expressed sequences. *In silico *sequence variation detection was performed using the dbSNP repository [22].

### Conditions for denaturing high-performance liquid chromatography (DHPLC)

Thirteen amplicons, covering 4588 bp of the *CHRM5 *locus were used, which covered the transcribed and splice junctions of exons 1 to 4. In addition, we screened 500 bp 5' to the transcription start sites of exon 1 and 700 bp 5' to exon 2, to examine the putative promoter region. DHPLC was performed on the Helix Automated DNA Analysis and Mutation Detection System (Varian Inc.) using the Helix DNA Column (Part No, CP28353; Varian, Inc) according to the manufacturers guidelines. Column temperature guidelines for DHPLC analysis were obtained using the online Stanford DNAMelt programme [23]. Oligonucleotide sequences, amplification conditions and oven temperatures used for screening are listed in Table 3. Putative heterozygous samples and a representative homozygous control were sequenced in both forward and reverse strands in order to characterise the polymorphism. Sequence chromatograms were reviewed manually to identify polymorphisms.

**Table 3:** List of oligonucleotide primers, PCR conditions and oven temperatures used, for amplification and DHPLC mutational analysis of the human muscarinic acetylcholine receptor M5 gene.

			PCR Conditions			
					
Fragment Name	Primer Sequence Forward	Primer Sequence Reverse	Tm (°C)	Mg++ (mM)	Amplicon Size (bp)	Tm (DHPLC)
5'Exon 1. b-1	catctgctcctttctttcttcc	tgagaaatcctggttgttcct	59	2.5	469	56,58
Exon 1. b-1	ttctgaaatacgaacaagcaga	gcacgagcagatttaatttca	58	2	449	51,55,56
5'Exon 2. a-1	gggtcttgctatgtcatccag	ccttatgggaccactgttgtaaa	60	2	387	60,55.5
5'Exon 2. a-2	tgcaccacataccattttgg	ccggacacaatatcactgtctt	60	2	437	51,53,57
Exon 2. a-1	cattttgctgaccctaaagacc	tggacgcactacctttaaaaac	60	2	299	58,59,60
Exon 3. b-1	taacagcccctttgtgaacg	cctgtgggagaaatgtggtt	60	2.5	418	57,60,61
Exon 4. a|b-1	acaagaaaatcatgctggtgtg	aggttcatggagaagattccaa	60	2	383	58,60
Exon 4. a|b-2	gccagctcaagacagttaacaa	agagaaactggatctggcactc	60	2	390	60,61
Exon 4. a|b-3	ttgggaagcggacagttc	tagctgctacaggtggtgagc	62	1.5	400	60,61,62
Exon 4. a|b-4	caattgggccaaagctgag	Ctttcgttgaaggttccttgg	60	2	374	59,60
Exon 4. a|b-5	atggctgtcacaaggtgaaaat	tgccagtacaacttctcttcca	60	2	394	58,59,60
Exon 4. a|b-6	ctttaagatgctgcttctctgc	tcattgttacccagtgtgcttc	60	2	384	57,59
Exon 4. a|b-7	gggagtttgccaatgaagtaaa	caagaccttcactcagatgtgg	60	1.5	375	55,57,59

Tm, annealing temperature for PCR amplification; Mg++, magnesium concentration for PCR amplification; bp, base pairs; Tm _DHPLC_, column temperature (°C) for denaturing high performance liquid chromatography (DHPLC) analysis.

### Genotyping of *CHRM5 *polymorphisms

Prior to association analysis allele frequencies were determined in the screening population. An arbitrary minimum minor allele frequency of 5% was used as a threshold for further analysis. All SNP genotyping was performed by restriction fragment length analysis. Experimental details including oligonucleotide sequences, amplification conditions and the restriction enzyme used for analysis are detailed in Table 4.

**Table 4:** List of oligonucleotide primers, PCR conditions and restriction enzymes used to discriminate genotypes and allele frequency in Victorian European Caucasian population of putatively functional human muscarinic acetylcholine receptor M5 gene polymorphisms.

			PCR Conditions			
					
SNP id	Primer Sequence Forward	Primer Sequence Reverse	Tm (°C)	Mg++ (mM)	Restriction Enzyme	Minor Allele Frequency
rs661968	gaagcattcaatgaatcttcaataagttct	atgcggtagatgaagactcc	55	2.5	MseI	C = 0.03
rs7162140	cattttgctgaccctaaagacc	tggacgcactacctttaaaaac	60	2	TaqI	T = 0.19
-257A>T	taacagcccctttgtgaacg	cctgtgggagaaatgtggtt	60	2.5	NlaIII	A = 0.03
rs2702309	ggcaaacaacttgacccacagaaaacga	cctgtgggagaaatgtggtt	60	2	MboII	A = 0.00
rs2702304	gaaaaaagtgactatgacaccccaaactag	tgattttcaccttgtgacagc	59	2	SpeI	T = 0.00
rs2576302	atggctgtcacaaggtgaaaat	tgccagtacaacttctcttcca	60 to 54 TD	2	DdeI	T = 0.00
rs2705353	atggctgtcacaaggtgaaaat	tgccagtacaacttctcttcca	60 to 54 TD	2	AlwNI	A = 0.00

Tm, annealing temperature for PCR amplification; TD, touchdown cycling conditions; Mg++, magnesium concentration for PCR amplification; touchdown, refers to -0.5°C change in Tm per amplification cycle.

### Statistical analysis

All traits were examined against genotype data under a dominant (AA or Aa > aa) and recessive (AA > Aa or aa) model. Categorical data, including nicotine dependence, alcohol dependence and cannabis dependence, were analysed using logistic regression. Quantitative traits including alcohol dose (units per week) and tobacco dose (cigarettes per week) were analysed using two-tailed Student's t-test to assess the equality of means across inheritance models.

No adjustment was made for multiple testing [14]

## Abbreviations

CB1R, Cannabinoid Receptor 1

cDNA, Complementary deoxyribonucleic acid

CHRNA7, Cholinergic Receptor Nicotinic Acid Type 7

CHRM5, Cholinerginc Receptor Muscarinic Type 5

CIDI, Composite International Diagnostic Interview

CPW, Cigarettes per week

Delta9-THC, Delta9-tetrahydrocannabinol

DNA, Deoxyribonucleic Acid

DSM-IV, Diagnostic Statistical Manual, Verson 4

DHPLC, Denaturing high-perfomance liquid chromatography

FTND, Fagerstrom Test of Nicotine Dependence

GABA, Gamma-aminobutyric acid

ICD-10, International Classification of Disase, Version 10

M5R, Muscarinic M5 receptor

nAChR, Nicotinic Acetylcholine receptor

NMDA, N-methyl-D-aspartate

OR, Odds Ratio

SEM, Standard Error of the Mean

SNc, Substantia nigra pars compacta

VAHCS, Victorian Adolescent Health Cohort Study

VAPSE, Variants Affecting Protein Structure or Expression

VTA, Ventral tegmental area

## Authors' contributions

RJLA conceived of the study, participated in study design, coordination, analysis and drafted the manuscript. MLM performed the laboratory experimentation, in silico analysis and assisited in data analysis and manuscript preparation. CAO participated in study design and coordination of sample collection of the VAHCS dataset. SCR participated in phenotype design. SAH coordinated data management and collection on the VAHCS. GCP participated in coordination of the VAHCS. All authors read and approved the final manuscript.
